# Enhancing the diagnostic accuracy of core needle biopsy in patients with lymphoproliferative disorders by an optimized protocol

**DOI:** 10.1007/s11547-025-01976-2

**Published:** 2025-04-01

**Authors:** Silvia Ferrari, Alessandra Weber, Paolo Marra, Paola Tebaldi, Chiara Pavoni, Anna Maria Barbui, Giuseppe Gritti, Ludovico Dulcetta, Francesco Saverio Carbone, Riccardo Muglia, Paola Anna Erba, Andrea Gianatti, Alessandro Rambaldi, Sandro Sironi

**Affiliations:** 1 Hematology and Bone Marrow Transplant Unit, ASST Papa Giovanni XXIII Hospital, Bergamo, Italy; 2 School of Medicine and Surgery, University of Milano-Bicocca, Milan, Italy; 3 Department of Radiology, ASST Papa Giovanni XXIII Hospital, Bergamo, Italy; 4 Department of Pathology, ASST Papa Giovanni XXIII Hospital, Bergamo, Italy; 5 Nuclear Medicine Unit, ASST Papa Giovanni XXIII Hospital, Bergamo, Italy; 6 Department of Oncology and Hematology, Università Degli Studi Di Milano, Milan, Italy

**Keywords:** Biopsy, Large-core-needle, Lymphoproliferative disorders, PET/CT, Ultrasonography, Interventional

## Abstract

**Purpose:**

Surgical excision biopsy of lymph nodes stands as the gold standard for histological characterization of lymphoproliferative disorders (LD). However, contemporary clinical practice increasingly leans toward core needle biopsy (CNB). This study seeks to explore the factors influencing the diagnostic yield of CNB in LD.

**Material and Methods:**

This unicentric retrospective study presents data from patients referred for suspicion of new or relapsing LD. All patients underwent image-guided CNB of the target lesion based on PET/CT findings. The primary endpoint was the diagnostic outcome, comparing the ability to achieve a definitive diagnosis according to international guidelines with CNB versus the necessity for subsequent excisional biopsy.

**Results:**

We enrolled 478 consecutive patients undergoing CNB, categorized into two cohorts. Cohort A comprised patients who underwent CNB using 18-20G full-core Menghini needles, with a median macroscopic fragment dimension of 1 cm. Cohort B included patients who underwent CNB with 16-18G semiautomatic guillotine needles, with a median macroscopic fragment dimension of 1.5 cm. In cohort A, the rates of diagnostic and non-diagnostic (or non-sufficiently detailed) CNBs were 95 (73%) versus 35 (27%), respectively. In cohort B, these rates were 299 (86%) versus 49 (14%).

**Conclusion:**

The type and size of the needle used for CNB, as well as the histologic variant of LD, emerged as factors influencing diagnostic yield and accuracy. Given the swiftness of CNB compared to surgical excision, optimizing this technique could streamline the diagnostic and therapeutic workflow for patients with suspected LD.

## Introduction

The diagnosis of lymphoproliferative disorders historically relies on tissue specimens sampled by surgical excision, which provides information about tissue architecture, overcoming the limitations of fine needle cytology. While surgical excision (SE) remains the gold standard for histological diagnosis as recommended by clinical guidelines from the European Society of Medical Oncology (ESMO) [1–3], the College of American Pathologists [4], and the National Comprehensive Cancer Network (NCCN) [5], in clinical practice fine needle aspiration cytology (FNAC) and core needle biopsy (CNB) performed with radiological image-guidance are more and more often used to expedite the diagnostic process [6]. While FNAC alone is deemed inadequate, CNB may represent an alternative when excisional biopsy is not feasible or safe despite caution must be taken in certain lymphoma subtypes, (i.e. Hodgkin lymphoma (HL) and follicular lymphoma (FL)), where CNB may yield insufficient material for accurate tissue architecture assessment and grading [2, 3]. Nevertheless, to date, comparison between the results of SE and CNB predominantly stem from single-center experiences [7] and retrospective series [8, 9]. Limited randomized trials and meta-analyses exist [10, 11], prompting experts to advocate for cautious interpretation of findings [12]. The most recent World Health Organization (WHO) Classification of Tumors of Hematopoietic and Lymphoid Tissue [13] identifies nearly 100 lymphoid malignancies, primarily defined by histologic findings. The incorporation of flow cytometry, karyotypic analysis, and molecular tests has significantly enhanced diagnostic accuracy. [1–12] In this study we are presenting our experience from real-world data on the use of CNB for the diagnosis of suspect lymphoma in a large cohort of patients. In particular, the data aim to assess the diagnostic accuracy of CNB, analyzing the critical factors influencing the diagnostic yield.

## Patients and methods

### Trial design

We retrospective assessed data from all patients consecutively referred to our haematological center with suspected lymphoproliferative disorder (LD) between January 1 2017 and April 30 2022.

The study was conducted in accordance with the principles of the Declaration of Helsinki [14]. All the patients signed informed consent for the CNB, for the storage of the samples in the biobank and, in case of confirmation of hematological diagnosis, for collection of clinical data in our Lymphoid Cancer Registry (NCT03131531).

The primary endpoint of the study was to assess the diagnostic yield of CNB. Specifically, we aimed to evaluate (1) the diagnostic accuracy for definitive LD characterization and (2) the impact of different needle types and sizes on CNB accuracy. As for the secondary endpoint, we sought to evaluate the rate of procedure-related complications.

**Fig. 1:**
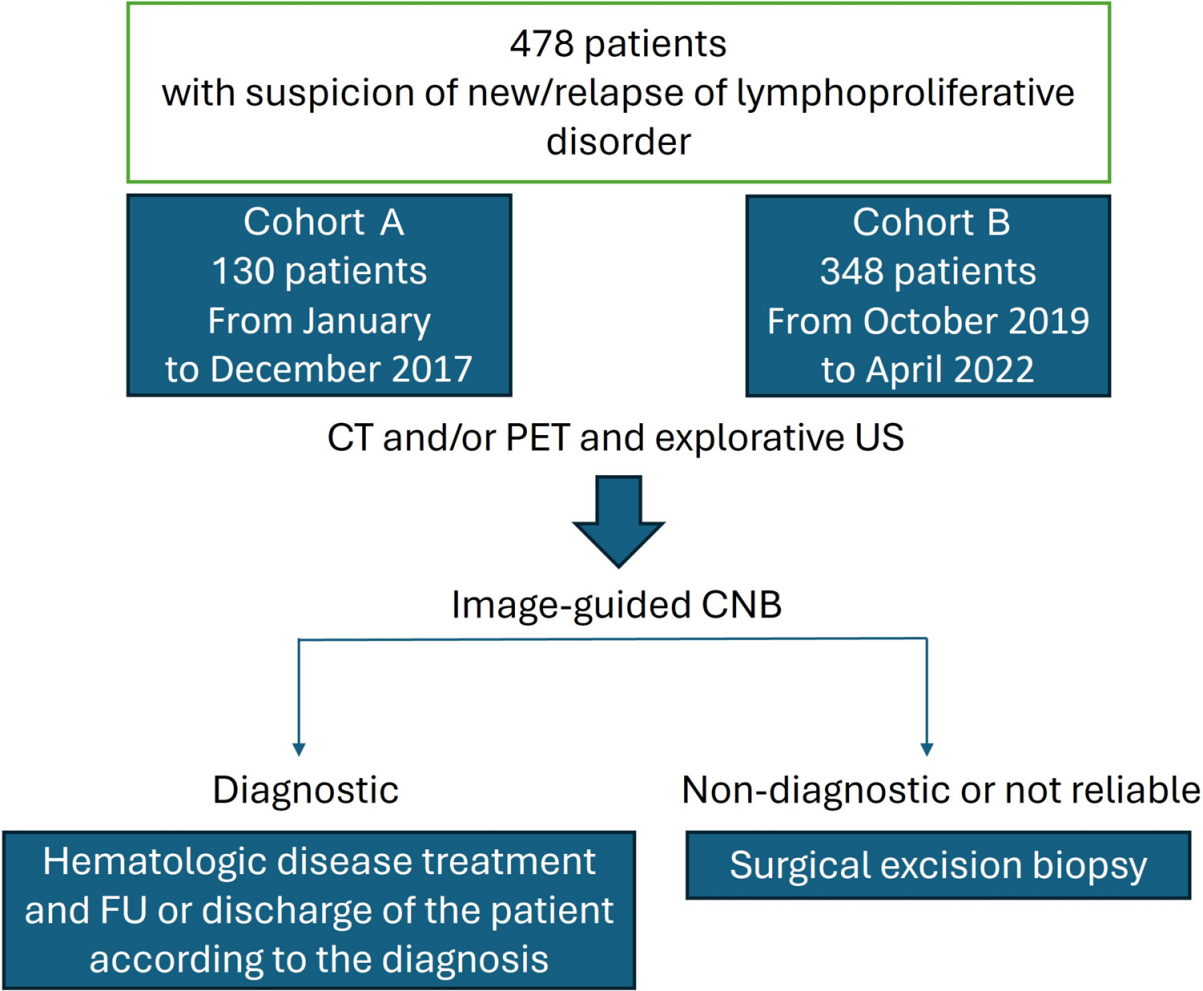
Flow diagram of the study; CNB, core needle biopsy

The design of the study is described in the diagram in Fig. 1.

All the patients underwent core needle biopsy (CNB) for disease characterization. Diagnostic yield was defined based on the pathologist’s confidence to provide a proper diagnosis, including histological subtype, grading, and the request of ancillary tests (e.g., fluorescence in situ hybridization, next-generation sequencing for specific mutations). If the pathologist and hematologist concurred on a histologic diagnosis consistent with the clinical phenotype after CNB analysis, no further diagnostic procedures were necessary. However, if CNB yielded an inconclusive or unfeasible diagnosis, treatment plans were retained until the patient underwent SE.

Furthermore, we performed a sub-analysis of two different patients cohorts based on the size of the core needles used. Cohort A included patients referred from January to December 2017, for which older smaller size needles were used; cohort B included patients referred from October 2019 to April 2022 for which we introduced the use of larger needle aiming to improve CNB accuracy. For each patient in both cohorts, clinical presentation, LD history, biopsy anatomical region, needle size and type, short-term procedure complications, sample size, and diagnostic outcome were examined (Table 1).

**Table 1:** Baseline characteristics of the study population and technical data about CNB

Characteristics	Cohort A	Cohort B
Period of enrolment	January 2017 to December 2017	October 2019 to April 2022
Number of patients	130	348
Male patients (%)	62	58
Median age (years)	61	64
Suspect new diagnosis vs relapse (%)	59	61
Needle type	Menghini full-core	Semiautomatic guillotine
Needle size (Gauge)	18–20	16–18
Median size of the tissue sample (cm)	1	1.5
Complications (according to CTCAE, 5.0.)	None	1 grade 1, short-term 3 grade 3

Patients diagnosed with a LD were treated or observed according to the institutional protocols and, follow-up visits were retrospectively reviewed to confirm consistency of the LD diagnosis. Patients with no need for immediate treatment were visited for follow-up 4 months after the diagnosis and every 6 months afterward. Patients were subjected to blood tests and physical examination at every check-up. At alternate follow-up visits, instrumental tests were performed, such as chest X-ray and abdominal ultrasound. Radiological tests were anticipated whenever there was a clinical suspicion of disease progression. If the suspicion was confirmed, the patients were subjected to a new complete work-up. Patients with other diagnoses were discharged and treated or observed accordingly.

### Study population

For the purpose of this study, 478 consecutive patients were enrolled; demographics and the main characteristics of patients are outlined in Table 1. Among the 130 patients in cohort A, 76 (58%) were referred with suspicion of a new diagnosis of LD, 49 (37%) with a suspected disease relapse of a previously known LD, and 5 (4%) with suspected progression/transformation from an indolent to an aggressive LD.

In cohort B, 348 patients included 213 (61%) referred with suspicion of a new diagnosis of LD, 106 (31%) with a suspected relapse of a previously known LD, and 29 (8%) with suspected progression/transformation from an indolent to an aggressive LD.

### Biopsy technique

**Fig. 2:**
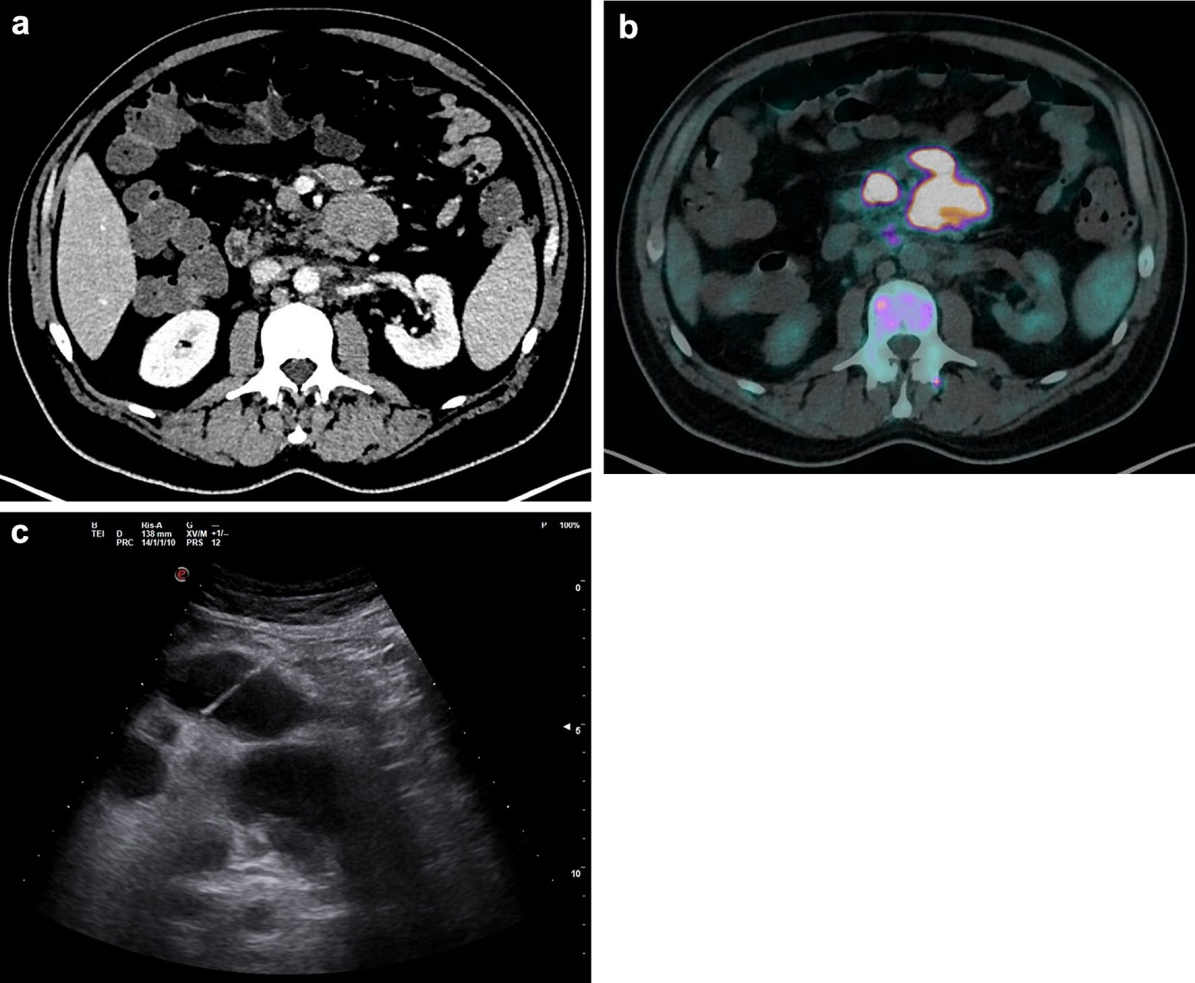
Core needle biopsy diagnosis of large B cell lymphoma in a patient with large mesenteric lymph nodes. Preoperative contrast-enhanced CT (**A**) and [18F]FDG PET/CT (**B**) images were evaluated to define the most favorable target for percutaneous biopsy. A transabdominal ultrasound-guided percutaneous core needle biopsy (**C**) was performed with a 18G semiautomatic guillotine needle and provided the definitive diagnosis

**Fig. 3:**
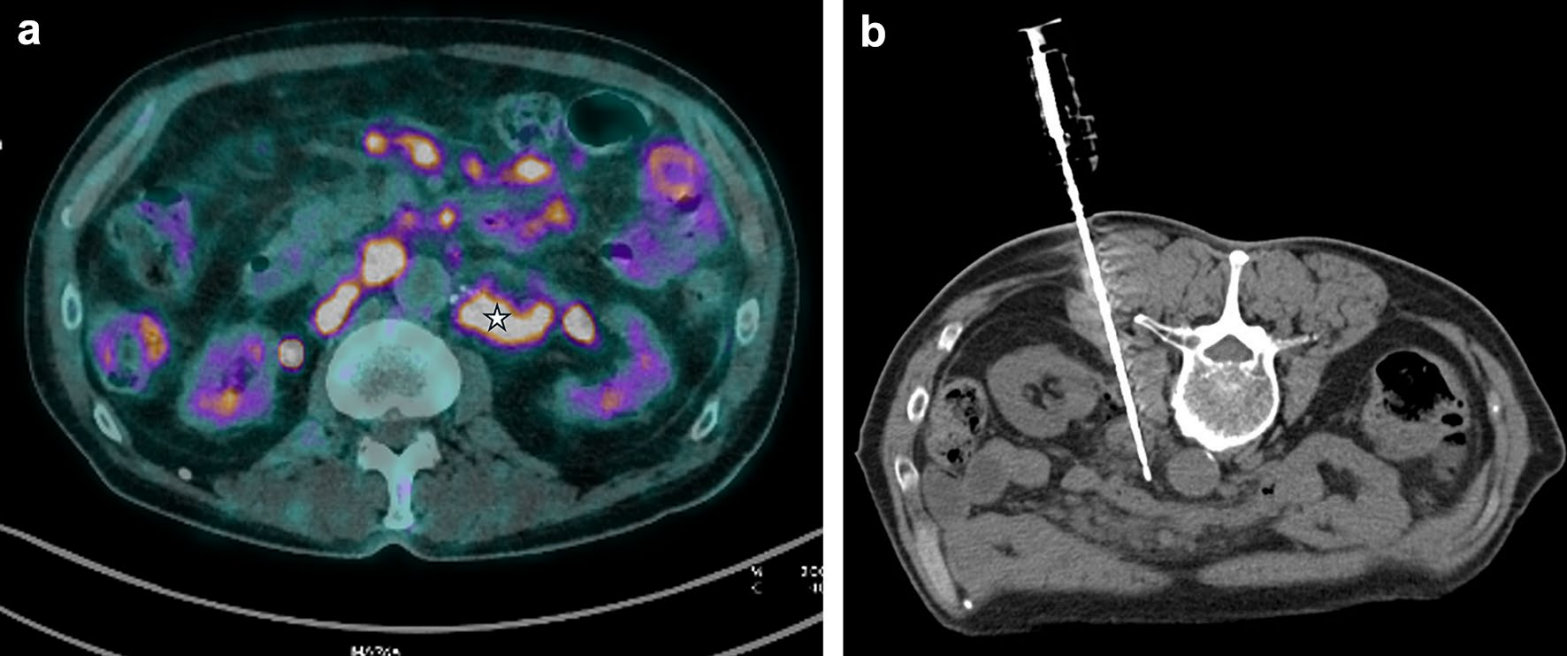
Core needle biopsy of enlarged retroperitoneal lymph nodes in a patient with Whipple’s disease. Preoperative [18F]FDG PET/CT (**A**) image shows multiple enlarged lymph nodes in the retroperitoneal space with a maximum SUV of 12.6. Left para-aortic lymph nodes are chosen as the safest biopsy target (star). With the patient lying in a prone position a CT-guided translumbar coaxial biopsy was performed: image (**B**) shows multiplanar reformation of the needle trajectory

Before CNB procedures, the radiologists defined the target lesion reviewing the available computerized tomography (CT) or fluorine-18-deoxyglucose positron emission tomography/computerized tomography ([^18^F]FDG PET/CT) imaging as the most representative lesion at the easiest accessible site. Further, an exploratory patients’ assessment with ultrasound (US) was performed to evaluate procedure feasibility and safety. CNBs were conducted under either US or CT guidance, based on the optimal biopsy tract, lesion size, morphology, and anatomical location to minimize complication risks[15]. For example, in case of superficial lymphadenopathies they were punctured directly under US guidance. On the contrary, CT guidance was chosen for deep retroperitoneal targets using a translumbar route. In case of large peritoneal, retroperitoneal, mediastinal, and lung masses, either US or CT guidance was employed according to the operator’s preference, (Figs. 2 and 3).

US-guided procedures were performed in a sterile fashion using a free-hand technique. A 2% lidocaine hydrochloride local anesthetic was injected along the percutaneous biopsy tract. Needle size was determined based on the operator’s preference and preliminary risk assessment, with 18–20 Gauge full-core Menghini needles employed in the cohort A of patients and 16–18 Gauge semiautomatic guillotine needles used in the cohort B. At least three needle passes were performed to obtain three tissue cores, with up to six passes or more conducted for superficial targets with easy access. After each pass, the tissue core was transferred into formalin, and the needle was washed into sterile saline to collect cells for cytofluorimetry.

For deeply located targets and spleen biopsies, the coaxial technique was utilized, with gel foam slurry injected through the cannula for track embolization. After needle removal, compression was applied to the body surface for approximately 10 min.

Patients who underwent superficial biopsies were observed briefly in a recovery room, while those undergoing deep or solid organ biopsies were observed for at least three hours. Depending on clinical judgment, a blood count may have been performed before discharge. In accordance with recommended standard operating procedures[16], antiplatelet and anticoagulant therapies were only withheld for deep biopsies and resumed the day after the procedure if no bleeding occurred. Pre-procedural blood counts and coagulation status were checked only for patients undergoing deep or solid organ biopsies. Biopsy-related complications were graded according to the Common Terminology Criteria for Adverse Events (CTCAE) version 5.0.

### Histologic preparations

Specimens were fixed in 10% buffered formalin, embedded in paraffin, and sectioned at 3 μm thickness as per routine procedure. These sections were stained with hematoxylin–eosin. Immunohistochemistry and Epstein-Barr virus-encoded RNA in situ hybridization were conducted when necessary, using the BOND-III system from Leica. Immunohistochemistry procedures were performed either with the BOND-III Leica or with the Dako Autostainer.

## Statistical analysis

The data are presented as numbers with percentages for categorical variables and as medians for continuous variables for each patients’ cohort. Logistic regressions were employed to assess factors influencing the diagnostic rate, and odds ratios (OR) with 95% confidence intervals (95% CI) were reported. All P values were two-sided, and a significance level of 5% was established. Statistical analyses were conducted using R software (version 4.3.0).

## Results

The median time between the initial hematological evaluation and the first radiological assessment was 7 days while the median time from radiological assessment to CNB was 5 days. An expedited processing within 48 working hours was done for clinically urgent cases (*n* = 12). Following CNB, the median time for the pathological report was 10 days. Overall, the diagnostic process took a median of 23 days (range 15–33 days) to be completed.

Tables 2 and 3 summarize the distribution of the involved anatomical regions for CNBs in cohort A and B, respectively. The median needle dimension used for CNBs was 20 Gauge (range 18–21) and 16 Gauge (range 16–20) and the median macroscopic dimension of fragments was 1 cm and 1.5 cm in cohort A and B, respectively.

**Table 2:** CNB sites and diagnostic outcomes of patients in cohort A

Site of CNB	Neck	56
	Inguinal region	28
	Axillary region	22
	Abdominal region	18
	Mediastinum	3
	Head	2
	Upper limb	1
Diagnostic rate of CNB	Diagnostic CNBs	95 (73%)
	Non diagnostic CNBs	9 (7%)
	CNBs deemed not sufficiently detailed	26 (20%)
Diagnostic reports of reliable CNBs	Diffuse large B cell lymphoma	24
	Hodgkin lymphoma	21
	Follicular lymphoma	17
	Aggressive B cell lymphomas	7
	Low grade B lymphomas	5
	T cell lymphoma	5
	Inflammation and/or necrosis	7
	Solid tumor	7

**Table 3:** CNB sites and diagnostic outcomes of patients in cohort B

Site of CNB	Neck	149
	Abdominal region	61
	Inguinal region	47
	Axillary region	44
	Mediastinum	15
	Head	10
	Liver/spleen	8
	Muscle	4
	Digestive tract	4
	Upper limb	3
	Inferior limb	2
	Bone	1
Diagnostic rate of CNB	Diagnostic CNBs	299 (86%)
	Non diagnostic CNBs	33 (9%)
	CNBs deemed not sufficiently detailed	16 (5%)
Diagnostic reports of reliable CNBs	Diffuse large B cell lymphoma	71
	Aggressive B cell lymphomas	30
	Mantle cell lymphoma	10
	Hodgkin lymphoma	48
	Follicular lymphoma	42
	Low grade B cell lymphomas	28
	T cell lymphoma	11
	Inflammation and/or necrosis	29
	Solid tumor	30

### CNBs diagnostic yields

Among the 130 CNBs performed in cohort A, 95 (73%) were considered diagnostic (final diagnosis is shown in Table 2), whereas the remaining 35 patients (27%) required additional diagnostic analyses because of non-diagnostic samples (9 procedures, 7%) or inconsistent results (26 procedures, 20%). Of these patients, 32/35 underwent SE demonstrating the presence of indolent lymphomas (11 cases), aggressive B cell lymphomas (7 cases), peripheral T cell lymphoma (3 cases), HL (3 cases) and benign conditions (5 cases). In 3/130 (0.02%) cases, no pathological condition was identified. Of this cohort, the final diagnostic rate of CNBs with a diagnosis of LD was 79/106 (74%).

Among the 348 CNBs performed in the cohort B, 299 (86%) were considered diagnostic (for specific diagnosis see Table 3). In 49 patients (14%) additional diagnostic analyses were requested because of non-diagnostic samples (33 procedures, 9%) or inconsistent results (16 procedures, 5%). In 40/49 patients, SE was performed resulting HL (9 cases) and FL (6 cases), diffuse large B cell lymphoma (DLBCL, 3 cases) and benign processes (2 cases). No pathological condition was present in 7 cases. In cohort B the final diagnostic rate of CNB in the 281 cases of LD was 85.4% (240/281).

Accordingly, the diagnostic accuracy (Table 4) was higher in cohort B compared to cohort A, with an odds ratio (OR) of 2.25 (95% confidence interval [CI] 1.37–3.67, *p* = 0.0012). The best diagnostic yield was achieved in cases of aggressive B cell lymphomas and carcinomas. None of the patients’ baseline characteristics significantly influenced the diagnostic rate in both cohorts nor the site of the biopsy (deep vs superficial) (OR 1.59, 95% CI 0.52–6.91, *p* = 0.4701). The only factor significantly associated with higher diagnostic rate was the dimension of the histological sample (OR 2.73, 95% CI 1.28–6.32, *p* = 0.0127).

**Table 4:** Factors affecting the diagnostic rate between cohort A and B

Characteristics	Number of diagnoses (%)	OR (95% CI)	P
Cohort			
(A) 2017	95 (73.1)	1	
(B) 2019–2022	299 (85.9)	2.25 (1.37–3.67)	0.0012
Site			
Superficial	303 (82.1)	1	
Deep	91 (83.5)	1.1 (0.63–2)	0.7409
Dimension of histological fragment		2.28 (1.57–3.36)	< .0001
Diagnosis			
Reactive process	26 (65)	1	
Solid tumor	37 (92.5)	6.64 (1.93–30.95)	0.0058
Hodgkin lymphoma	69 (80.2)	2.19 (0.94–5.08)	0.0678
infection	10 (90.9)	5.38 (0.89–104.01)	0.1259
B cell aggressive lymphoma	142 (91.6)	5.88 (2.48–14.13)	0.0001
B cell indolent lymphoma	95 (75.4)	1.65 (0.75–3.52)	0.1999
T cell lymphoma	15 (78.9)	2.02 (0.6–8.13)	0.2819

For the 118 patients that were diagnosed with DLBCL, the histologic fragments were big enough to perform not only the standard pathological diagnostic tests, but also the additional cytogenetic analyses (MYC, BCL2 and BCL6 rearrangements) that allow to properly identify high risk patients (so-called double-hit DLBLC patients when MYC rearrangement is present together with either BCL2 and BCL6). Of all the patient that sign informed consent ad hoc it was possible also to conserve a sample in our biobanca.

### Procedure-related complications

In cohort A, no complications were observed. In cohort B we observed one grade 1 (G1) vagal crisis and three grade 3 (G3) complications, accounting for 0.8% of cases. These included a rectus abdominis hematoma, a duodenal perforation, and a hemoperitoneum resulting from splenic bleeding, all conservatively managed.

## Discussion

Although surgery traditionally served as the gold standard for histological diagnosis of LD by excising the entire suspect lymphadenopathy with no therapeutic role except for rare isolated indolent lymphoma locations, current clinical practice has increasingly relied on FNAC and CNB. These techniques are favored for their speed, safety, and minimally invasive nature [6, 17–26]. In particular, CNB offers clear advantages, especially in case of deep-seated lesions for which it can be performed safely and without the need for general anesthesia, compared to SE. Furthermore, CNB histologic reports are frequently available earlier than SE, reducing the need for additional tissue sampling [8, 9, 27, 28]. This approach streamlines the diagnostic process, reducing patient discomfort and risk.

A notable aspect of our study is the strict adherence to high diagnostic accuracy standards, which considered unreliable all cases in which histological lymphoma subtype, grading, or cytogenetic information were lacking. Moreover, this is the first study comparing the diagnostic accuracy among two different CNB needle on LD fashion, demonstrating how larger core needles can bring a favorable outcome, avoiding lymph node excision.

Being image-guided, CNB may be anticipated to be more precise than SE in identifying the target lesion, i.e. pathological lymph node with the most predictive imaging features [8]. Despite these potentials, SE is still considered superior to CNB due to the advantages of providing samples for the assessment of the whole lymph node architecture, a key element for the differential diagnosis and for grading specific lymphoma subtypes (i.e., FL) [9], for ancillary tests such as cytogenetic analysis and for biobanking. Recently, retrospective studies [8, 9] and meta-analyses [10, 11] confirmed the superiority of SE over CNB in terms of diagnostic accuracy [12]. In particular, a French study [29] reviewing 32,285 cases registered in the French Lymphopath network, reported a good diagnostic rate—according to the WHO—for both SE and CNB, with higher diagnostic accuracy for SE in terms (98.1%) as compared to CNB (92.3%).

The potential benefits and harms obtained by increasing needle size in CNB have been rarely investigated [30]. Although the architecture of the lymph node still remains not evaluable due to the nature of CNB sampling, the sample size and the number of fragments achievable with increased needle size could provide enough material to fulfill the need of ancillary tests and for long-term conservation and biobanking. Indeed, in our experience using percutaneous CNB as the primary approach to diagnose LD we were able to reach a reliable diagnosis in 85.4% when using semiautomatic or automatic guillotine-type needles for nodal sampling under real-time imaging guidance. The needle type changed in the two cohorts for two reasons: first, the original and skilled team of interventional radiologists who performed the majority of cohort A procedures retired over time. The interventional radiology team renewal coincided with the introduction of new type of needles and with the request from the Pathology Unit of larger tissue specimens.

Following the results of our study, we recommend the use of such protocol for CNB. We obtained the best diagnostic accuracy in case of aggressive B cell lymphomas which resulted to be the forms that most likely benefit form a minimally invasive diagnosis with CNB. Our hypothesis is that aggressive lymphomas are usually identified even if the cells are few because the cellular morphology and immunohistochemistry are sufficient for diagnosis. Low grade lymphomas and Hodgkin more often requires an evaluation of the lymph node architecture. In the case of Hodgkin’s lymphomas, the problem is the frequent absence of evaluable Red Stemberg cells in a little specimens. Surprisingly, we also obtained a good diagnostic rate and characterization in case of HL, whereas T cell lymphomas and non-aggressive B cell lymphomas had the lower diagnostic rates. Among the patients in which CNB was not diagnostic in both cohorts, FL peripheral T cell lymphoma, classic HL, and nodular lymphocyte-predominant HL and benign processes were most often diagnosed. This data is not surprising when considering that FL, HL, and peripheral T cell lymphoma are among the most challenging to diagnose, given the importance of the architectural structure and proportion between anatomical regions of the lymph node. Indeed, guidelines [1–3, 5] strongly recommend SE lymphadenectomy for these histologic subtypes to achieve a thorough classification of specific subtypes. Accordingly, SE should be preferred as a primary option whenever these LD subtypes are suspected and the clinical conditions are favorable.

As known for other organs, the decrease of needle gauge improves the probability of a diagnostic biopsy, but entails a higher risk of procedure-related complications [31, 32]. Consistently, in our population we found a different gauge-related complication incidence among cohort A and B (0 vs 0.8%), without need for major treatment. Due to very low values, we address this finding more to the lymph node location than to gauge size, as all the complicated procedures were requested for deep sites.

Interestingly, a small proportion of patients with suspected LD turned out to be affected by solid tumors that were appropriately diagnosed by CNB. In this subgroup the advantage of CNB over SE was even more appreciated, avoiding the invasiveness of a useless and potentially harmful surgical approach.

Furthermore, a special mention should be deserved to cases of histological transformation, where an indolent lymphoma transitions into a more aggressive form. While SE would have the advantage of removing the lymph node in its entirety for complete evaluation, CNB offers the unique opportunity of direct targeting the lymph node with the higher standardized uptake values based on pre-procedural [^18^F]FDG PET/CT scan. In these cases proper image-based procedure is critical [33]. Indeed, in case of discordance between the imaging findings and histopathology, the clinician should consider a second targeted biopsy, even if the initial CNB report suggests an indolent form to ensure thorough evaluation and the exclusion of focal areas of transformation within the lymph node.

### Limitation of the study

Our study has several limitations. Firstly, we did not address factors such as changes in operators over time and the availability of improved equipment for pathological and cytological assessment, which may have influenced the results. Additionally, we did not perform a comparative analysis between patients who underwent primary SE (10 throughout the whole study duration), which resulted in a lack of data about the feasibility of CNB as a primary approach. Furthermore, we were not able to perform a match comparison between CNB and SE in most patients, as CNB was sufficient to reach a diagnosis in the majority of cases. This lack of matching precluded the determination of positive/negative predictive values of CNB compared to SE. Moreover, the lack of standardization of biopsy sample quality assessment and the subjectivity of pathology reporting limits prompt generalizability of our findings.

## Conclusions

Our study demonstrated the feasibility and value of CNB as first-line diagnostic procedure in patients with suspected LD, shortening patients’ workflow and limiting the necessity of SE. The optimization of technical aspects are key to improve the sampling quality to be compliant with the requirements of current international guidelines.

## Abbreviations

LD: Lymphoproliferative disorders
FNAC: Fine needle aspiration cytology
CNB: Core needle biopsy
SE: Surgical excision
HL: Hodgkin lymphoma
FL: Follicular lymphoma
DLBCL: Diffuse large B cell lymphoma
